# Condition, not eyespan, predicts contest outcome in female stalk-eyed flies, *Teleopsis dalmanni*

**DOI:** 10.1002/ece3.1467

**Published:** 2015-04-08

**Authors:** Eleanor Bath, Stuart Wigby, Claire Vincent, Joseph A Tobias, Nathalie Seddon

**Affiliations:** Department of Zoology, Edward Grey Institute, University of OxfordOxford, OX1 3PS, UK

**Keywords:** Armaments, female–female competition, male–male competition, mutual ornamentation, sexual selection, social selection, status signaling, *Teleopsis dalmanni*

## Abstract

In contests among males, body condition is often the key determinant of a successful outcome, with fighting ability signaled by so-called armaments, that is, exaggerated, condition-dependent traits. However, it is not known whether condition and exaggerated traits function in the same way in females. Here, we manipulated adult condition by varying larval nutrition in the stalk-eyed fly, *Teleopsis dalmanni*, a species in which eyespan is exaggerated in both sexes, and we measured the outcome of contests between females of similar or different body condition and relative eyespan. We found that females in higher condition, with both larger bodies and eyespan, won a higher proportion of encounters when competing against rivals of lower condition. However, when females were of equal condition, neither eyespan nor body length had an effect on the outcome of a contest. An analysis of previously published data revealed a similar pattern in males: individuals with large relative eyespan did not win significantly more encounters when competing with individuals of a similar body size. Contrary to expectations, and to previous findings in males, there was no clear effect of differences in body size or eyespan affecting contest duration in females. Taken together, our findings suggest that although eyespan can provide an honest indicator of condition, large eyespans provide no additional benefit to either sex in intrasexual aggressive encounters; body size is instead the most important factor.

## Introduction

Exaggerated ornamental traits in males are widely viewed as the most conspicuous product of sexual selection (Darwin 1871; Andersson 1994). These secondary sexual characters are used to attract mates and often play a role in mediating competition with rivals over mating opportunities (Andersson 1994). The majority of studies on exaggerated traits have focused on males, neglecting the wide variety of taxa in which females also possess similar traits (“mutual ornamentation”: Kraaijeveld et al. 2007; Tobias et al. 2012). The existence of exaggerated traits in females has traditionally been attributed to a genetic correlation between the sexes, where sexual selection favoring male trait expression results in a correlated response in females (Darwin 1871; Lande 1980). Theory (Lande 1980) and empirical work (Wilkinson 1993; Price 1996; Potti and Canal 2011) have shown that genetic correlations between the sexes exist in certain situations. However, recent studies indicate that female exaggerated traits can also be under strong selection (e.g., Watson and Simmons 2010; Rosvall 2011; Mahr et al. 2012). A crucial next step is to establish the form of this selection, particularly whether it is similar to, or different from, that acting on males (Tobias et al. 2012).

Studies investigating adaptive explanations for female ornamentation tend to focus on male mate choice and often neglect other explanations, such as female competition over ecological or social resources (Kraaijeveld et al. 2007; but see Watson and Simmons 2010; Midamegbe et al. 2011). Nonetheless, access to these resources is a key factor for female fitness, suggesting that traits that increase resource acquisition ability are potential targets for selection (Berglund et al. 1993). In “classical” sexually selected mating systems, males often fight over and monopolize access to females, resulting in dramatic differences between winners and losers. Females, on the other hand, often compete over access to resources which are more likely to increase survival or fecundity or be used to rear offspring, such as favorable nesting sites (Rosvall 2008) or food to provision offspring (Robinson and Kruuk 2007; Watson and Simmons 2010). Winners in female competition may be able to acquire larger shares of such resources, but are unlikely to be able to monopolize them, perhaps leading to incremental increases in fitness over their competitors rather than the “winner takes all” results in males (Clutton-Brock 2009).

Condition, defined as “the pool from which resources are allocated” (Rowe and Houle 1996), plays a key role in male intrasexual competition, where proxies of condition such as body size or sexually selected trait size are often key predictors of contest outcome and duration (McCann 1981; Dugatkin and Biederman 1991). The role of condition in female competition is, however, much less clear. In some species, individuals in higher condition are more directly successful in female competition or acquire higher social status (Petrie 1988; Griggio et al. 2010; Watson and Simmons 2010; Cain and Ketterson 2012), but in others, condition appears to play little or no role in deciding intrasexual encounters (Dugatkin and Biederman 1991; Draud 2004; Elias et al. 2010). If condition is important in determining the outcome of female competition over resources which influence fitness, we would expect that traits that signal an individual’s condition or competitive ability to be used in intrasexual contests (Berglund et al. 1993). We would also expect that the ability to determine a rival’s size or fighting ability will not only affect contest outcome but also the duration of intrasexual contests (Parker 1974; Maynard Smith and Parker 1976). Contest duration should be determined by differences in resource holding potential (“RHP”: Parker 1974) between competitors – the larger the difference, the shorter the contest (Parker 1974). Traits which enable the quick recognition of RHP or competitor ability should help to minimize costs by shortening duration or preventing individuals from participating in contests they have no chance of winning (Parker 1974). One implication of these hypotheses is that exaggerated traits in females may often be “armaments,” which are used as weapons or signals of dominance in intrasexual contests, rather than “ornaments,” which are used to attract mates. We follow the previous literature in using this definition of “armament” to include signals (rather than just weapons), as it allows us to draw useful parallels with “ornaments” (Berglund et al. 1996).

While single traits functioning as both ornament and armament are widespread in males (Berglund et al. 1996), the situation in females is less clear (Tobias et al. 2011). The question of whether exaggerated traits could function as female armaments and mediate female–female competition has received little attention (Berglund et al. 1996; Griggio et al. 2010; Kekäläinen et al. 2010), in part because exaggerated traits and their fitness consequences are less easily quantified in females (Tobias et al. 2011). Female competition can be difficult to study because few contests are decisively resolved, leading to unclear dominance patterns, as well as less dramatic escalation of behaviors than in males (Nilsen et al. 2004; Clutton-Brock and Huchard 2013). Exaggerated condition-dependent traits in females have also often been neglected in studies of intrasexual competition because they fail to show the same levels of exaggeration and heightened condition dependence as their male homologs (Kraaijeveld et al. 2007). Females are argued to experience a trade-off between fecundity and trait expression, which hampers the evolution of trait exaggeration (Fitzpatrick et al. 1995). This lack of exaggeration has been used to justify a lack of research interest into the potential signaling qualities of female traits (Amundsen 2000). However, although female traits are, in general, not as exaggerated as male traits, this is no reason why they cannot function as honest, condition-dependent signals. In fact, the proposed trade-off between fecundity and trait expression may be the very mechanism which ensures honesty in female traits (Simmons and Emlen 2008).

To explore the role of condition and female traits in intrasexual competition, we investigated the function of an exaggerated trait in the stalk-eyed fly, *Teleopsis dalmanni*. In *T. dalmanni*, males possess large sexually selected eyestalk ornaments and females possess smaller but nonetheless exaggerated eyestalks of unknown function (De la Motte and Burkhardt 1983; Baker and Wilkinson 2001; Al-khairulla et al. 2003). It has recently been suggested that there is male mate choice for females with longer eyestalks, suggesting that there are selective pressures acting on female eyespan (Cotton et al. 2014). Individuals vary in eyestalk length, measured as the span across both eyestalks, and it is easily quantifiable in both sexes. To test the importance of condition and eyespan, we experimentally manipulated female larval nutrition to generate variation in condition, and hence, body size and eyespan, and conducted intraspecific contests over food, measuring the outcome of contests in terms of their duration and proportion of encounters won. We used body length as our proxy for condition (David et al. 1998; Cotton et al. 2004). As eyespan is also used as a measure of condition in *T. dalmanni*, we aimed to evaluate whether eyespan solely functions as a measure of body length or whether it can provide additional information about the condition of an individual (David et al. 1998; Cotton et al. 2004). It has been suggested that eyestalks are more sensitive to condition than body size traits and may therefore provide additional, or more accurate, information during rival assessment in intrasexual competition (Wilkinson and Dodson 1997; Panhuis and Wilkinson 1999). To test this, we investigated whether eyespan explained any additional variance in contest outcome after controlling for larval diet treatment and body size. Furthermore, to evaluate similarities between the sexes in the use of exaggerated traits in intraspecific competition, we reanalyzed data from a classic study on male competition in *T. dalmanni* using analogous methods (Panhuis and Wilkinson 1999). Panhuis and Wilkinson used randomly selected individuals, representing the variance available in a laboratory population but did not manipulate condition in their experiment. While larval diet treatment is the main cause of variation in condition in females in our experiment, pre-existing standing variation in body size of unmanipulated males from a laboratory population is used as an index of condition in Panhuis and Wilkinson (1999), meaning that male body size is not identical to a larval diet treatment. The differences in experimental design, and evaluation of condition, mean that although we cannot therefore directly compare our results, qualitative comparisons should highlight any major differences between the sexes in how they use exaggerated traits in intrasexual competition.

If eyespan is a condition-dependent signal of quality mediating intrasexual competition in female *T. dalmanni,* we predicted the following:

1. Flies in higher condition (i.e., those from less restricted larval diet treatments) will have larger eyespans
2. Flies in higher condition will win a higher proportion of agonistic encounters than flies in lower condition.
3. As the difference in condition between competitors increases, contest duration will decrease.
4. When flies are matched for condition, individuals with larger eyespan relative to their body condition will win a higher proportion of encounters.

We also hypothesized that prediction 4 would also hold true for all males from Panhuis and Wilkinson (1999).

## Materials and Methods

### Study species

*Teleopsis dalmanni* is a sexually dimorphic stalk-eyed fly found in Southeast Asia (De la Motte and Burkhardt 1983; Swallow et al. 2005). The eyestalks of males are generally longer, thinner and flatter than those of females and can be up to one and a half times male body length (Swallow et al. 2005; Worthington et al. 2012). Male eyespan is highly condition dependent, increasing dramatically with increased larval nutrition and body size (David et al. 1998; Cotton et al. 2004); female eyespan is also condition dependent, though to a lesser degree (David et al. 1998; Cotton et al. 2004). Previous studies showed that males with larger eyespan relative to their body length are more likely to win in male–male competition (Panhuis and Wilkinson 1999; but see Brandt and Swallow 2009), and females prefer to roost and mate with males with larger relative eyespan (Burkhardt and de la Motte 1988; Hingle et al. 2001). Females also engage in physical contests generally over food, both in the wild and in the laboratory (Burkhardt and de la Motte 1983; Al-khairulla et al. 2003). Although female contests resemble those of males, where individuals line up face-to-face and strike each other with their forelegs (Panhuis and Wilkinson 1999), it is unknown whether condition plays any role in determining contest outcome, and whether eyespan explains any further variation in contest outcome (Al-khairulla et al. 2003).

### Fly rearing

Flies used were from the laboratory population of *T. dalmanni* founded in 1993 with wild-captured individuals from Gombak, Malaysia (Cotton et al. 2004; Rogers et al. 2006). We kept all flies in cages at 25°C with 70% humidity on a 12:12 h cycle (light: dark). Population size has been kept high (>200 individuals) to minimize inbreeding (Cotton et al. 2004; Rogers et al. 2006). The flies were fed ad libitum with blended sweet corn, which was replaced twice a week. All experiments were conducted in 2013, with initial treatments (block 1) replicated later in the year (block 2).

### Manipulation of condition and eyespan

Larval nutrient availability determines adult body size and eyespan and has been established as a determinant of condition in *T. dalmanni* (David et al. 1998). In block 1, batches of 20 eggs were collected from population cages and placed in Petri dishes lined with damp cotton wool (the same quantity for each treatment). Following thresholds proposed by David et al. (1998), we generated differences in female eyespan by assigning eggs randomly to one of three diet manipulations: 0.015 g (restricted), 0.03 g (medium), and 0.06 g (fully fed) corn per egg. In block 2, we transferred batches of 15 eggs, each randomly assigned to one of the same three provisioning treatments. Previous studies have shown that individuals raised on more restricted larval diets (e.g., flies on the restricted treatment vs. flies on the medium treatment) eclose into smaller adults which have shorter eyestalks than flies from less restricted larval diet treatments (e.g., flies on the fully fed treatment are on a less restricted diet than those on the medium treatment) (David et al. 1998; Cotton et al. 2004). Flies took approximately 3 weeks to eclose. One week after eclosion, we separated flies according to sex and dietary treatment to ensure virginity. Flies were then housed in same-sex and same-treatment group cages (10–20 individuals per 2 L cage in block 1 and 40–60 per 10 L population cage in block 2, resulting in similar population densities across treatment blocks). In total, 526 females were used in this experiment.

### Female contests

When females reached sexual maturity (4 weeks after eclosion), we anaesthetized flies by placing them on ice and took photographs of all individuals lying on their thoracic spines at 7.5× magnification using a Canon EOS 600D SLR camera mounted on a Leica M80 microscope. We then used the program ImageJ (Rasband 1997) to measure eyespan and body length following landmarks used by Wilkinson (1993).

Flies were removed from group cages 24 h before being used in a contest and placed in separate Petri dishes containing damp cotton wool but no food. Depriving flies of food was to increase their motivation to fight and increase our chances of observing contests, as flies were fighting over food (blended sweet corn) in the contest. In block 1, we placed a dot of acrylic paint (red or yellow) on females’ thoraxes 24 h before they were used in a contest (Al-khairulla et al. 2003). Unfortunately, the flies removed the paint used to mark them, so we used body length to distinguish individuals. We used ImageJ to measure individuals from still images taken from each contest video and matched these to the measurements taken under the microscope, enabling us to identify individual flies. In block 2, to aid identification of individual flies, a small part of one wing (left or right) was cut close to the tip of the wing before flies were placed in their individual Petri dishes (Chenoweth et al. 2007). Flies do not appear to use their wings during fights, although there is the potential for flies to use the wings for balance (Al-khairulla et al. 2003). Half (50%) of the flies in each treatment had their left wing clipped; the rest had their right wing clipped. Flies were randomly assigned to treatments, but were always matched against a fly which had the opposite wing clipped.

There were six fight treatments (all possible combinations), based on larval dietary manipulations: fully fed versus fully fed (FF), fully fed versus medium (FM), fully fed versus restricted (FR), medium versus medium (MM), medium versus restricted (MR), and restricted versus restricted (RR). “F” females tended to develop into the largest individuals, “R” the smallest, and “M” intermediate. Females from each group were randomly assigned to a fight treatment on the day of the contest but were never matched with a female from the same cage. There were between three and five cages for each treatment in each block. The contest arena consisted of a Petri dish, with one damp cotton wool pad as lining and a small dab of blended corn in the center. Contests were held over 2 h following lights-on with females matched for age. Flies were aspirated into the arena at the same time and allowed 5 min to acclimatize. The arena was then filmed for 20 min. Each fly was used in only one contest.

### Scoring contests

Contest videos were analyzed using JWatcher (http://www.jwatcher.ucla.edu/; Blumenstein et al. 2006). We scored four variables to quantify the intensity and result of contests: the number of encounters, the duration of each encounter, the fly initiating each encounter, and the contest outcome. For each individual, there were three possible results for each encounter: win, loss, or no result. The winner of each interaction was scored as the fly that did not retreat or turn away first (Panhuis and Wilkinson 1999; Al-khairulla et al. 2003). If it was unclear which fly turned away first, the encounter was scored as a “no result.” Videos were scored blind with respect to the female treatment, although in size-mismatched treatments, there was no way to conceal obvious size differences.

### Statistical analysis

Generalized linear models (GLMs) with a quasibinomial error distribution were used to test the effects of larval diet treatment, body length, and eyespan on the “proportion of encounters won” and “contest duration.” A quasibinomial distribution was used to adjust for overdispersion. To allow for nonindependence of individuals from the same dyad, dispersion was calculated using the number of dyads to calculate degrees of freedom, rather than the number of individuals, and doubling the sum of the residuals from the fitted model (McCullagh and Nelder 1989). “Proportion of encounters won” was used as the primary response variable and was calculated as the number of encounters won by the focal individual divided by the total number of decided encounters (where there was a winner and loser). To test predictions related to contest duration, and to ensure model residuals were normally distributed, the natural log of total fight duration and the natural log of mean fight duration were used as response variables in general linear models. Block was originally included as a fixed effect in all models, but had no significant effects so was removed and is not included in the models reported here. Data on male–male competition came from a previous study on the role of eyespan in male stalk-eyed flies, including *T. dalmanni* (Panhuis and Wilkinson 1999). Males and females were analyzed in separate models as the data came from different experiments. The first set of female analyses focused solely on differences between larval diet treatments, without including body length or eyespan as factors. This allowed us to test our prediction that flies from less restricted larval treatments would win more encounters.

To control for differences in diet treatment and body size between competitors in a second set of models, treatments were reclassified into “matched” and “unmatched” body size treatments. Treatments were “matched” by pairing females with an individual from the same larval diet treatment, so that both individuals were similar in body size (range of differences: 0.002–1.397 mm). We investigated the importance of investment in eyespan relative to body length by analyzing only the “matched” treatments, including larval diet and body length as covariates in the analysis, thus allowing us to evaluate the effect of eyespan after taking into consideration the effect of larval diet treatment and body length (Cotton et al. 2009). For males, where there was no larval diet manipulation, we used all data from Panhuis and Wilkinson (1999), so only body length and eyespan were included in the model. All data were analyzed in R version 2.15.2 (R Core Team 2012).

## Results

### Prediction 1: Flies in higher condition (i.e., those from less restricted larval diet treatments) will have larger eyespans

**Figure 1:**
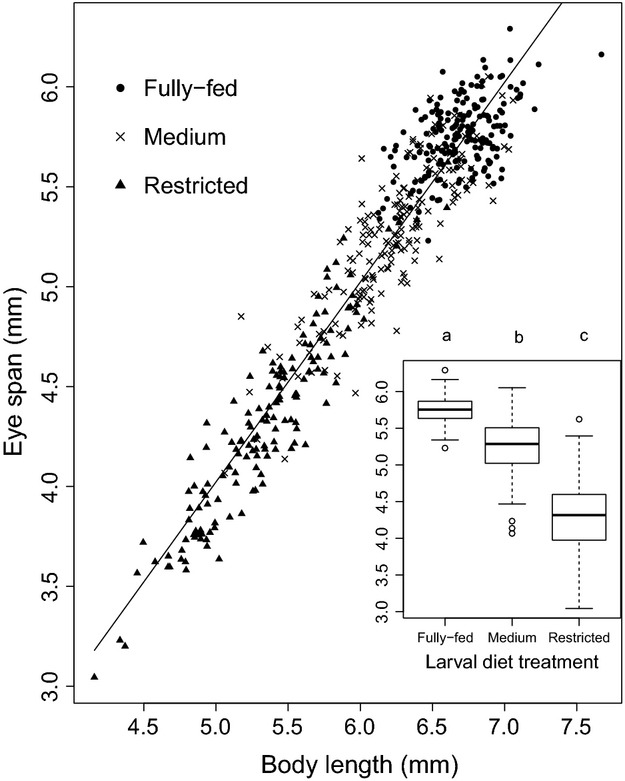
Relationship between eyespan and body length in female *Teleopsis dalmanni*. Circles = fully fed diet treatment (*n* = 193), crosses = medium larval diet treatment (*n* = 178), and triangles = restricted larval diet treatment (*n* = 154). The linear regression line is shown (eyespan = 0.999 × body length − 0.97, *R*^2^ = 0.92). The inset boxplot reflects the differences in mean between the treatments, with post hoc Tukey tests indicated by letter subscripts. Different letters indicate significant differences between treatments.

In support of this prediction, we found that larval diet treatment had a significant effect on both eyespan and body length (eyespan: *F*_2,522_ = 723.5, *P* < 0.0001 and body length: *F*_2,522_ = 636.9, *P* < 0.0001; Inset Fig.1). Females from the fully fed treatment had the largest eyespans (5.75 ± 0.01 mm) and body lengths (6.69 ± 0.02), while flies from the restricted treatment had the smallest (ES: 4.32 ± 0.04, BL: 5.34 ± 0.04). Flies from the medium treatment were intermediate in size (ES: 5.25 ± 0.03, BL: 6.23 ± 0.03). In addition, there was a significant positive correlation between female eyespan and body length across all treatments (*F*_1,522_ = 5727, *P* < 0.0001, eyespan = body length − 0.978, *R*^2^ = 0.92; Fig.1).

### Prediction 2. Flies in higher condition will win a higher proportion of agonistic encounters than flies in lower condition

**Figure 2:**
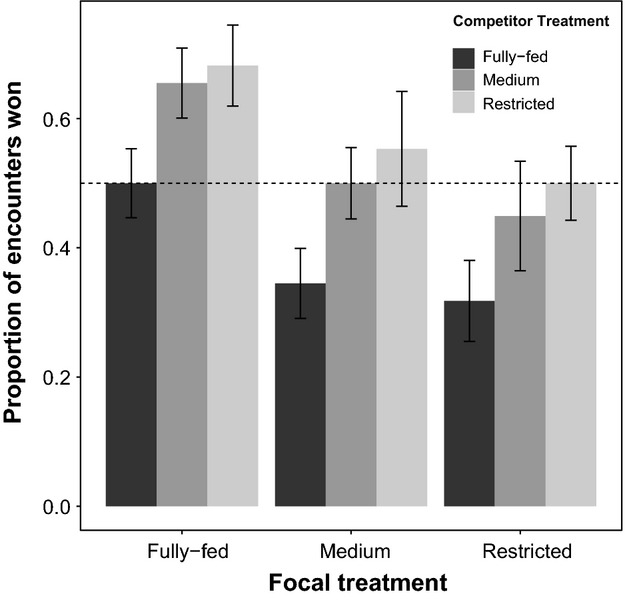
Effect of diet treatment on proportion of encounters won in females. Individuals from less restricted diet treatments (i.e., fully fed and medium) won a higher proportion of encounters when competing against individuals from more restricted larval diet treatments (i.e., medium and restricted). Columns represent means, with error bars indicating standard errors. The dotted horizontal line indicates a proportion of 0.5 – when the focal fly wins the same number of fights as its competitor.

In support of this prediction, we found that diet treatment of the focal fly had a significant effect on the proportion of encounters won, with females of higher condition winning a greater proportion of encounters (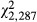  = 12.12, *P* = 0.028; Fig.2). Competitor diet treatment also had a significant effect on the proportion of encounters won with focal individuals winning a higher proportion of encounters when competing against individuals from more restricted diet treatments (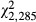 = 20.07, *P* = 0.002). However, there was no significant interaction (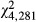  = 0.57, *P* = 0.99). In other words, flies from less restricted diet treatments (i.e., fully fed and medium) won a higher proportion of encounters than females from more restricted diet treatments (i.e., restricted).

### Prediction 3. As the difference in condition between competitors increases, contest duration will decrease

**Figure 3:**
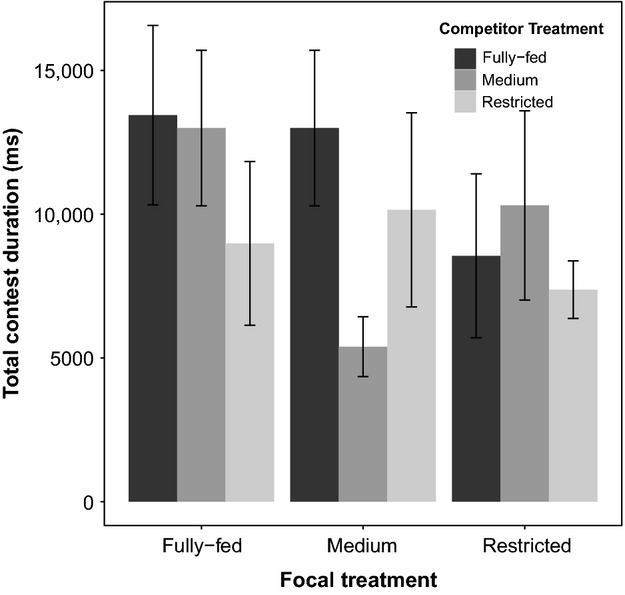
Effect of diet treatment on total contest duration in females. There was no discernible pattern due to larval diet treatment in total contest duration in females. Columns represent means, with error bars indicating standard errors.

We did not find support for this prediction. Specifically, we found that diet treatment of the focal fly had a significant effect on total contest duration: females of higher body condition fought for longer (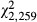  = 208,525, *P* = 0.038; Fig.3). However, there was no significant effect of competitor diet treatment (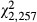  = 23,249, P = 0.694) or the interaction between focal and competitor treatment on contest duration (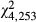  = 108,310, *P* = 0.492). When focusing on instances where flies fought individuals from the same larval diet treatment, there was a significant difference in total contest duration between treatments, where females from the “fully fed” treatment fought for longest, followed by those from the “restricted” treatment, with females from the “medium” treatment fighting for the least amount of time (*F*_2,252_ = 3.59, *P* = 0.029). However, the only significant difference between treatments in post hoc tests was between “medium” and “restricted” treatments (Tukey multiple comparison: restricted – medium – *t* = 2.67, *P* = 0.022), where females from the “restricted” fought for longer than those from the “medium” treatment.

### Prediction 4. When flies are matched for condition, individuals with larger eyespan relative to their body condition will win a higher proportion of encounters in:

#### Females

We found no support for this prediction in females. There was no significant effect of larval diet treatment (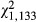  = 0, *P* = 0.99), body length (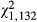  = 0.53, *P* = 0.576), or eyespan after controlling for body length on the proportion of encounters won (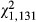  = 0.25, *P* = 0.7; Fig. S1A). Furthermore, there was also no significant interaction between diet treatment and body size (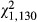  = 3.56, *P* = 0.149) or between diet treatment and eyespan (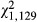  = 0.57, *P* = 0.564). In other words, when a female faced a competitor of the same level of condition, possessing larger eyestalks relative to body length did not increase the proportion of encounters an individual won.

#### Males

We also found no support for this prediction in males, contrary to previous findings. Specifically, we found that while there was a significant effect of body length on the proportion of encounters won (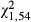  = 51.9, *P* = 0.009), with larger individuals winning a higher proportion of encounters, there was no effect of eyespan after controlling for body length (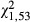  = 1.02, *P* = 0.714; Fig. S1B). In other words, males with longer eyestalks relative to body length did not win a higher proportion of encounters.

## Discussion

In this study, we found that eyespan is strongly correlated with body length (our proxy for condition) in female *T. dalmanni*, suggesting there is the potential for eyespan to function as an armament in intrasexual competition. However, body length rather than eyespan was the key determinant of contest outcome, with larger females winning a higher proportion of encounters than those in lower condition. However, contrary to our predictions and previous findings in males, body length appeared to play no role in determining contest duration. Eyespan relative to body length was not associated with success in aggressive interactions when fighting similarly sized same-sex rivals, in either males or females.

Eyespan is closely linked to body length (i.e., it is condition dependent) in both male and female *T. dalmanni* (David et al. 1998; Cotton et al. 2004). As contest outcome is determined by body length, it seems logical that condition is the underlying factor determining contest outcome. If condition is important in determining contest outcome, it is possible that eyespan functions as an armament to signal body size and/or fighting ability. In the wild, *T. dalmanni* form linear aggregations on long, thin root hairs at dusk (De la Motte and Burkhardt 1983). When both males and females fight, they orient themselves face-to-face, with their eyestalks parallel to each other (Panhuis and Wilkinson 1999; Al-khairulla et al. 2003). This would make it difficult to judge body size directly, but allows the easy assessment of eyespan. This method of fighting, which appears to be closely linked to the narrow, vertical nature of their aggregation environment (Burkhardt and de la Motte 1983; De la Motte and Burkhardt 1983), could lead females to use eyespan as the main means by which to assess body size and fighting ability of their opponent. Detectable differences in eyespan may indicate differences in condition and/or fighting ability that could influence the outcome of a contest.

Once we controlled for body length, we found that eyespan did not significantly influence contest outcome. This does not mean that eyespan does not function as an armament, but merely that after body length is controlled for, eyespan explains no more variance in contest outcome. By controlling for body length, we hoped to test whether eyespan merely functions as a measure of body length or whether it represents a “wider range of condition factors that act independently of body size” (Cotton et al. 2004). For neither females nor males does eyespan appear to reflect additional factors of condition that influence intrasexual competition. In other words, when competing against an individual of the same body length, having more exaggerated eyestalks (which should indicate higher condition) does not appear to give an individual an advantage. This is what we would expect whether there was a trade-off between eyestalk length and other fitness traits, such as fecundity. The prediction would be that females with traits exaggerated beyond what is necessary to adequately signal condition should have reduced fitness, because resources invested in “extra” eyestalk length could have been spent on increasing body size, and hence increasing fecundity (Fitzpatrick et al. 1995). This trade-off may not just restrict the mean eyestalk length of the population but also prevent cheating by smaller individuals (Simmons and Emlen 2008). If individuals overly invest in eyestalks to win a higher proportion of encounters, they will be able to produce fewer offspring than those that invest at the “right” level (Chenoweth et al. 2007).

Eyestalks in stalk-eyed flies have been used as a prime example of intrasexual competition leading to extreme trait exaggeration in evolutionary textbooks, but our results do not support this interpretation. As long as eyespan accurately reflects body size, it should be able to function as an armament, regardless of the level of trait exaggeration. Male eyespan is under sexual selection by means of female mate choice, as females prefer males with longer eyestalks (Burkhardt and de la Motte 1988). Therefore, it is possible that male eyespan has become more exaggerated due to female choice and has remained an accurate signal of condition to be used in male competition, but it seems unlikely to have been selected for its function in male intrasexual competition (“ornament–armament” hypothesis: Small et al. 2009).

To more rigorously examine whether *T. dalmanni* actually use eyespan as a signal in intrasexual competition, we would need to uncouple eyespan and body length, which is experimentally challenging. In one study where eyespan relative to body length was artificially selected for, flies selected for increased relative eyespan also showed an increase in body size, demonstrating the difficulty in trying to decouple eyespan and body length (Wilkinson 1993). In our current study, we were able to manipulate body length (and therefore condition), but not alter the relationship between body length and eyespan. Further work manipulating eyestalk length through altering imaginal discs during development, or manually transferring eyestalks, to create large flies with small eyespans and vice versa, may give a better indication of whether and how *T. dalmanni* use eyespan as a signal of condition in intrasexual competition (Warren and Smith 2007; Brandt and Swallow 2009). As condition can be affected by both larval and adult diet, it would be informative in future studies to manipulate adult diet and larval diet, giving a better understanding of what role condition plays in determining the outcomes of intrasexual competition.

The trend that flies of larger body size, that is, those with higher resource holding potential (“RHP”: Parker 1974), win more fights concurs with previous findings in males of the same species (Panhuis and Wilkinson 1999; Brandt and Swallow 2009), as well as with females in other species (Petrie 1988; Watson and Simmons 2010; Crowhurst et al. 2012). Contest duration, however, did not show the expected trend. There was no indication that differences in size between competitors affected contest duration. According to theory, competitors that are extremely different in size (or any other indicator of RHP) should be able to quickly determine who is larger and more likely to win a fight, resulting in smaller individuals giving up sooner against a larger opponent to minimize costs (Parker 1974; Maynard Smith and Parker 1976; Hammerstein and Parker 1982). A fight between a smaller individual and a larger individual should therefore take less time than a contest between two similarly sized individuals. This theory assumes that there is some method of assessing a rival and that individuals are able to judge the relative strength of the two competitors (Fawcett and Mowles 2013). Our finding that contest duration is not affected by relative size differences between female competitors suggests that there may be no rival assessment in female *T. dalmanni*. Individuals may instead “decide” how long they compete for based on their own energy reserves and the perceived resource payoff value (“RPV”: Draud 2004) of the contested resource. It has been suggested that in male *T. dalmanni,* contest duration is determined by loser body size rather than the difference in size between the two competitors (Brandt and Swallow 2009). If duration is determined by losers reaching an internal threshold sooner and retiring from the contest, condition is still the primary determinant of contest outcome, but is functioning in a more indirect way than what has been envisaged under mutual assessment theory (Taylor and Elwood 2003; Fawcett and Mowles 2013). If condition acted this way in females (i.e., individuals persisted until they reached their internal energy threshold), we would have expected to see flies in higher condition fighting for longer against flies in similar condition and contests decreasing in duration as the condition of competitors decreased (Taylor and Elwood 2003). We did observe flies from the “fully fed” larval diet treatment fighting for longer against opponents matched for condition, but “medium” treatment flies fought for less time than “restricted” treatment flies. “Restricted” females fighting for longer than “medium” females could indicate that “restricted” flies have an increased threshold for fight length because they are so short of resources that they must continue to fight despite their lack of energy reserves.

A second possible explanation for this lack of expected trend in contest duration is that individuals may value the contest resource differently, leading to different levels of motivation and willingness to compete (Bishop et al. 1978; Enquist and Leimar 1987). The individual that values the resource more should be willing to expend more energy and/or to risk more to secure the resource (Draud 2004). Females may be more variable in their resource valuation than males, due to characteristics that change over time, such as mating status, fertility, and age (Clutton-Brock and Huchard 2013). Both the costs and benefits of engaging in competition may therefore change for an individual female over time (Bowler et al. 2002; Papadopoulos et al. 2009; Seebacher et al. 2013). It seems unlikely that there was a great deal of variation between females in perceived resource value in our experiment, however, as females were all virgins of the same age and underwent the same period of food deprivation before their trials. It is, however, possible that this starvation period affected females from different treatments differently – females in lower condition may have been more strongly affected than those in higher condition with the internal resources to withstand a period without food. An increased desire for food in the lower condition flies could lead them to fight for longer against larger opponents than they would have if they valued the resource equally. Despite their increased motivation, however, these lower condition flies may still have been unable to overcome the size disparity between the competitors, leading to our observation of flies in better condition winning a higher proportion of encounters.

## Conclusion

Female *T. dalmanni* fight over food, and these contests are primarily determined by condition. Eyespan may function as an honest indicator of condition, but having larger eyestalks relative to body length does not appear to provide any additional predictive power for the outcome of intrasexual competition. The same appears to be true in males, despite the large differences in trait exaggeration between the sexes. It is possible that female exaggerated traits serve as armaments in deterring rivals from initiating intrasexual contests over ecological resources, but having higher relative eyespan confers no competitive advantage during such contests. Further work on female competition and the use of condition-dependent traits can help us to better understand the adaptive value of exaggerated traits in females and explain the understudied phenomenon of mutual ornamentation.

## Supporting Information

**Figure S1.** The effect of eyespan controlled for body length on proportion of encounters won.
