# Supplementary figures and images for: Condition, not eyespan, predicts contest outcome in female stalk-eyed flies, Teleopsis dalmanni
Source: Ecol Evol. 2015 Apr 8;5(9):1826–36. doi: 10.1002/ece3.1467 (PMC4485964; doi:10.1002/ece3.1467)

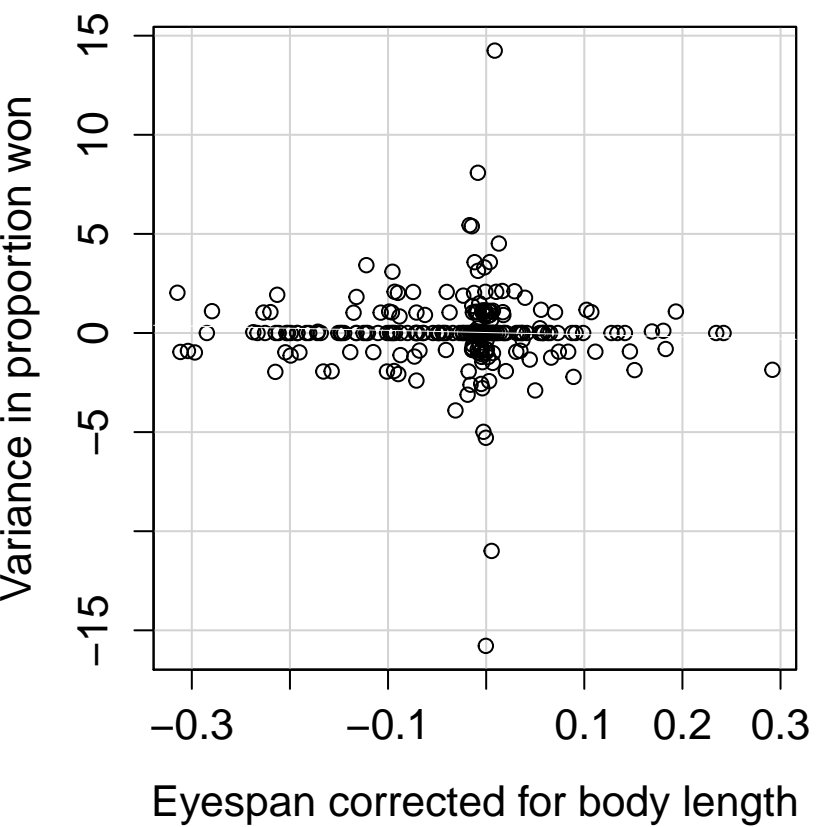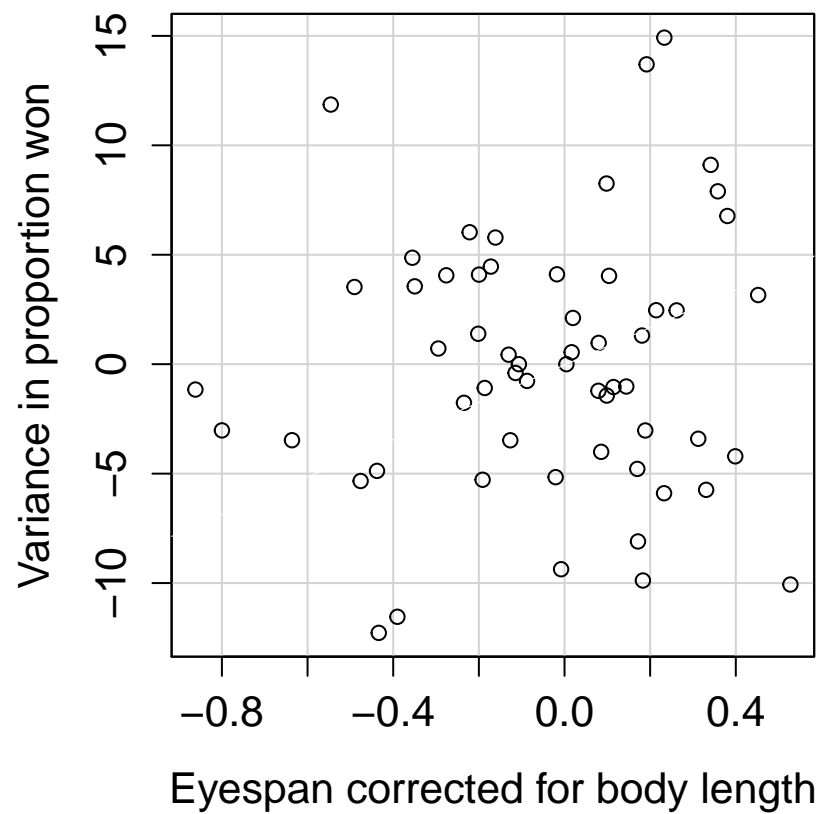

Supplement: Supplementary file 1 [file ece30005-1826-sd1.pdf]
