# Supplementary material for: Condition, not eyespan, predicts contest outcome in female stalk-eyed flies, Teleopsis dalmanni
Source: Ecol Evol. 2015 Apr 8;5(9):1826–36. doi: 10.1002/ece3.1467 (PMC4485964; doi:10.1002/ece3.1467)
Supplement: Supplementary file 2 [file ece30005-1826-sd2.docx]

**Supplementary information**

**Supplementary Figure S1: The effect of eyespan controlled for body length on proportion of encounters won**

The unexplained residuals of proportion of encounters won regressed on body size (i.e. the variance in proportion won not explained by body size) plotted against the residuals of eyespan regressed on body size (i.e. the variance in eyespan not explained by body size). These are shown in a) females matched for body size and b) all males from Panhuis & Wilkinson 1999. There was no significant effect of eyespan in either sex after being corrected for body length on the proportion of encounters an individual won.
